# Genetic Variants of Fatty Acid Amide Hydrolase Modulate Acute Inflammatory Responses to Colitis in Adult Male Mice

**DOI:** 10.3389/fncel.2021.764706

**Published:** 2021-11-22

**Authors:** Haley A. Vecchiarelli, Robert J. Aukema, Catherine Hume, Vincent Chiang, Maria Morena, Catherine M. Keenan, Andrei S. Nastase, Francis S. Lee, Quentin J. Pittman, Keith A. Sharkey, Matthew N. Hill

**Affiliations:** ^1^Neuroscience Graduate Program, Cumming School of Medicine, University of Calgary, Calgary, AB, Canada; ^2^Hotchkiss Brain Institute, Cumming School of Medicine, University of Calgary, Calgary, AB, Canada; ^3^Mathison Centre for Mental Health Research and Education, Cumming School of Medicine, University of Calgary, Calgary, AB, Canada; ^4^Department of Cell Biology and Anatomy, Cumming School of Medicine, University of Calgary, Calgary, AB, Canada; ^5^Department of Psychiatry, Cumming School of Medicine, University of Calgary, Calgary, AB, Canada; ^6^Snyder Institute for Chronic Diseases, Cumming School of Medicine, University of Calgary, Calgary, AB, Canada; ^7^Department of Physiology and Pharmacology, Cumming School of Medicine, University of Calgary, Calgary, AB, Canada; ^8^Department of Psychiatry, Weill Cornell Medical College, New York, NY, United States

**Keywords:** endocannabinoids, colitis, cytokines, amygdala, inflammation

## Abstract

Cannabinoids, including *cannabis* derived phytocannabinoids and endogenous cannabinoids (endocannabinoids), are typically considered anti-inflammatory. One such endocannabinoid is *N*-arachidonoylethanolamine (anandamide, AEA), which is metabolized by fatty acid amide hydrolase (FAAH). In humans, there is a loss of function single nucleotide polymorphism (SNP) in the FAAH gene (C385A, rs324420), that leads to increases in the levels of AEA. Using a mouse model with this SNP, we investigated how this SNP affects inflammation in a model of inflammatory bowel disease. We administered 2,4,6-trinitrobenzene sulfonic acid (TNBS) intracolonically, to adult male FAAH SNP mice and examined colonic macroscopic tissue damage and myeloperoxidase activity, as well as levels of plasma and amygdalar cytokines and chemokines 3 days after administration, at the peak of colitis. We found that mice possessing the loss of function alleles (AC and AA), displayed no differences in colonic damage or myeloperoxidase activity compared to mice with wild type alleles (CC). In contrast, in plasma, colitis-induced increases in interleukin (IL)-2, leukemia inhibitory factor (LIF), monocyte chemoattractant protein (MCP)-1, and tumor necrosis factor (TNF) were reduced in animals with an A allele. A similar pattern was observed in the amygdala for granulocyte colony stimulating factor (G-CSF) and MCP-1. In the amygdala, the mutant A allele led to lower levels of IL-1α, IL-9, macrophage inflammatory protein (MIP)-1β, and MIP-2 independent of colitis—providing additional understanding of how FAAH may serve as a regulator of inflammatory responses in the brain. Together, these data provide insights into how FAAH regulates inflammatory processes in disease.

## Introduction

Cannabinoids typically confer anti-inflammatory effects and provide protection against peripheral inflammatory illnesses, such as inflammatory bowel diseases (IBD) (Picardo et al., 2019; Osafo et al., 2021). Phytocannabinoids, such as Δ^9^-tetrahydrocannabinol, derived from the *Cannabis sativa* plant, exert their biological effects through interactions with the endocannabinoid (eCB) system. The eCB system is composed of the lipid signaling molecules, *N*-arachidonoylethanolamine/anandamide (AEA) and 2-arachidonylglycerol (2-AG); their respective primary biosynthetic [*N*-acylphospitidyl ethanolamine-phospholipase D (NAPE-PLD) for AEA and diacylglycerol lipase (DAGL) for 2-AG] and metabolizing enzymes [fatty acid amide hydrolase (FAAH) for AEA and monoacylglycerol lipase (MAGL) for 2-AG]; and two receptors, CB_1_ and CB_2_ (Katona and Freund, 2012). While both cannabinoid receptors are expressed on immune cells (Munro et al., 1993) and largely act to suppress inflammatory processes, CB_2_ is the receptor primarily responsible for the regulation of immune function while CB_1_ is the primary receptor in the brain. The specific mechanisms by which eCBs exert their anti-inflammatory effects include the inhibition of cell proliferation and migration, as well as through suppression of cytokine production (Parolaro, 1999; Nagarkatti et al., 2009).

Numerous studies show that elevating eCB signaling in chronic inflammatory diseases, such as IBD, is protective (Massa et al., 2004; Engel et al., 2008; Marques et al., 2008; Storr et al., 2010). Mice with genetic deletions of cannabinoid receptors have increased susceptibility to IBD (Massa et al., 2004; Storr et al., 2008a,b, 2009; Engel et al., 2010) and collagen-induced arthritis (Kinsey et al., 2011). Alternately, pharmacological inhibition or genetic deletion of FAAH, pharmacological inhibition of MAGL or activation of CB_1_ improves chemically induced colitis in rodents (Massa et al., 2004; Alhouayek et al., 2011; Fichna et al., 2014; Sałaga et al., 2014; Sasso et al., 2015; Shamran et al., 2017). Furthermore, in neurological inflammatory conditions, including excitotoxic injury (Marsicano et al., 2003), experimental autoimmune encephalitis (EAE) (Walter and Stella, 2004; Rossi et al., 2010; Pryce and Baker, 2012), neurodegenerative diseases (Rossi et al., 2010; Sánchez and García-Merino, 2012; Vázquez et al., 2015), head injury (Panikashvili et al., 2006; Tchantchou et al., 2014), aging (Marchalant et al., 2008), and ischemia (Hayakawa et al., 2008), there are neuroprotective effects of eCBs, primarily through reducing the expression of proinflammatory cytokines.

Inhibiting the degradation of AEA through the administration of FAAH inhibitors is typically anti-inflammatory. FAAH inhibitors or genetic deletion of *Faah* results in decreased responses to lipopolysaccharide (LPS) administration (Naidu et al., 2007) and reduced inflammation in a carrageenan model of acute inflammation (Holt et al., 2005), both via a CB_2_ receptor dependent mechanism. Increasing AEA signaling *in vivo* or *in vitro*, reduces levels of proinflammatory cytokines and other inflammatory mediators, such as nitric oxide, and increases anti-inflammatory cytokines (Puffenbarger et al., 2000; Chang et al., 2001; Facchinetti et al., 2003; Ortega-Gutiérrez et al., 2005; Tham et al., 2007; Correa et al., 2009, 2010). This also occurs within the central nervous system, where FAAH inhibition reduces the expression of LPS-induced proinflammatory cytokines in the hypothalamus (Kerr et al., 2012).

The amygdala is a brain region that is influenced by inflammation, with different inflammatory stimuli [i.e., rodent models of bacterial infection, systemic cytokine administration or disease models for multiple sclerosis (MS), colitis and arthritis] elevating excitatory neurotransmission within subnuclei of the amygdala, such as the basolateral nucleus (BLA), which is related to associated increases in anxiety-like behavior (Han and Neugebauer, 2004; Porcher et al., 2004; Welch et al., 2005; Chen et al., 2013; Jain et al., 2015; Acharjee et al., 2018; Li et al., 2018; Munshi and Rosenkranz, 2018; Zheng et al., 2021). Furthermore, elevated indices of amygdala activation are also observed in patients with gastrointestinal diseases, including, irritable bowel syndrome, and IBD (Naliboff and Mayer, 2006; Labus et al., 2009, 2013; Agostini et al., 2011; Fukudo and Kanazawa, 2011; Hubbard et al., 2011; Tillisch et al., 2011; Bao et al., 2015; Icenhour et al., 2015; Weaver et al., 2016; Fan et al., 2019). We previously showed in a rodent colitis model that the amygdalar eCB system is altered, and that these changes contribute to anxiety-like behavior in rats (Vecchiarelli et al., 2021). Given that FAAH inhibition ameliorates colitis-induced anxiety, and has the ability to suppress inflammatory processes, it remains possible that one mechanism by which elevated AEA signaling in the amygdala could reduce inflammation-associated anxiety is via the suppression of inflammatory cytokines.

Recently, a knock-in mouse model was developed for the common human single nucleotide polymorphism (SNP) mutation in *FAAH* (C385A; rs324420) (Dincheva et al., 2015). Individuals with this SNP have FAAH in which a conserved (C) proline (AA129) is substituted with a (A) threonine, which makes FAAH more susceptible to proteolytic degradation (Dincheva et al., 2015). In A allele carriers, there are reduced FAAH protein levels and activity, as well as an increase in AEA levels (Dincheva et al., 2015; Mayo et al., 2020). There is little known about the effect of this SNP on inflammatory processes. Given that inhibiting FAAH is anti-inflammatory, these FAAH C385A SNP mice represent an ideal model system to investigate the effects of elevating AEA on gut inflammation, peripheral and amygdala inflammatory levels in a manner that may shed light on disease vulnerability for humans who possess this SNP. Based on the established role of AEA signaling, inflammation and disease, we hypothesize that reduced FAAH activity (as seen in A carriers) will improve gut inflammation and reduce circulating and amygdalar cytokines.

## Materials and Methods

### Animals

These studies utilized young adult (7–9 week old), male FAAH C385A mice (Dincheva et al., 2015). These mice were derived from the line previously generated (Dincheva et al., 2015) and back crossed on a C57/Bl6J strain for over twenty generations. Mice were maintained in a specified pathogen free facility and obtained from in-house breeding, which crossed heterozygous males and heterozygous females. This breeding resulted in the following genotypes, all of which were utilized: wild type (CC), heterozygous (AC), and homozygous (AA) for the mutation. Genotyping was performed at the Hotchkiss Brain Institute Molecular Core Facility. Both the AC and AA groups exhibit reduced FAAH activity and increased AEA levels (Dincheva et al., 2015; Mayo et al., 2020). A carriers (i.e., AC and AA groups), as in human studies (Hariri et al., 2009; Dincheva et al., 2015; Gärtner et al., 2019; Lazary et al., 2019), were collapsed (collectively referred to as A, whereas CC are referred to as C) for analysis, as the magnitude of FAAH activity and AEA level changes are similar in both the AA and AC groups (Dincheva et al., 2015). Mice were kept on an 12:12 h light-dark cycle and had *ad libitum* access to food and water. All experiments were performed during the light phase. All experiments were approved by the Health Sciences Animal Care Committee of the University of Calgary and followed guidelines from the Canadian Council on Animal Care.

### Colitis Induction

Under brief isoflurane anesthesia, mice received an intrarectal bolus (approximately 3 cm proximal to the anus) of 2,4,6-trinitrobenzene sulfonic acid (TNBS) [Millipore Sigma, Darmstadt, Germany, #92822; 4 mg; 50% (vol/vol) in ethanol/water], via a plastic cannula. Control animals received the same volume of saline. Body weight was monitored daily. Analysis took place 3 days after TNBS administration, when inflammatory responses peak (Elson et al., 1996).

### Macroscopic Tissue Damage

Colons were removed and washed with ice-cold physiological saline (0.9%) and cut open longitudinally and macroscopically scored blindly for damage and inflammation. These scores were based on the presence or absence of adhesions and diarrhea and the degree of ulceration similar to those previously reported (Riazi et al., 2008; Vecchiarelli et al., 2021).

### Myeloperoxidase Activity

Following macroscopic tissue damage assessment, a sample of colon was excised, snap frozen, and stored at −80°C for later use in a myeloperoxidase (MPO) activity assay, as previously described (Sigalet et al., 2010; Vecchiarelli et al., 2021). Briefly, samples were homogenized in hexadecyltrimethylammonium bromide (HTAB; Sigma-Aldrich, Darmstadt, Germany, #H5882) in potassium phosphate buffer, homogenized using a 5 mm stainless-steel bead (Qiagen, Hilden, Germany, #69989) and TissueLyser LT bead homogenizer (Qiagen) for 10 min at 5 Hz and centrifuged for 10 min at 15871 × g at 4°C. Supernatant was added to hydrogen peroxide and o-dianisidine dihydrochloride in a 96 well-plate. Absorbance was measured at 450 nm, three times over 1 min (SpectraMax M Plate Reader, Molecular Devices, San Jose, CA, United States). MPO was expressed in milliunits per gram of wet tissue, 1 unit being the quantity of enzyme able to convert 1 μmol of H_2_O_2_ to water in 1 min at room temperature. Units of MPO activity per minute were calculated from a standard curve using purified peroxidase enzyme (Sigma-Aldrich, #M6908).

### Cytokine/Chemokine Multiplex Assay (Plasma and Amygdala)

As previously described (Vecchiarelli et al., 2016), plasma was separated from trunk blood following collection by centrifugation for 20 min at 10,000 × g at 4°C. Plasma was aliquoted and stored at −80°C. Amygdalae were micro-dissected (Gray et al., 2015; Vecchiarelli et al., 2016, 2021) and stored at −80°C prior to processing for cytokine ELISAs. Samples were placed in 10 μL/mg of homogenization buffer [150 mM sodium chloride, 2.5 mm magnesium chloride, 5 mg/500 mL aprotinin and cOmplete™ protease inhibitors (Millipore Sigma, #11836145001)]. Tissue homogenization occurred with mechanical disruption using a 5 mm stainless steel bead and TissueLyser LT bead homogenizer for 2 min at 50 Hz. Homogenized samples were run through a 0.22 μm spin filter tube (Millipore Sigma, #UFC30GVNB) for 4 min at 12,000 g at 4°C. Total protein quantification was assayed using a Pierce bicinchoninic acid (BCA) assay according to the manufacturer’s protocol (Thermo Fisher Scientific, Waltham, MA, United States, #23225). Samples were diluted to a final protein concentration of 500 μg/mL.

Eotaxin [C-C motif chemokine ligand (CCL)11], granulocyte colony stimulating factor (G-CSF), granulocyte monocyte colony stimulating factor (GM-CSF), interferon (IFN)γ, interleukin (IL)-1α, IL-1β, IL-2, IL-3, IL-4, IL-5, IL-6, IL-7, IL-9, IL-10, IL-12 (p40), IL-12 (p70), IL-13, IL-15, IL-17A, IFNγ inducible protein (IP)-10 (C-X-C motif chemokine ligand 10; CXCL10), KC (CXCL1), leukemia inhibitory factor (LIF), LPS-induced CXC (LIX), monocyte chemoattractant protein (MCP)-1 (CCL2), macrophage colony stimulating factor (M-CSF), monokine induced by IFNγ (MIG), monocyte inflammatory protein (MIP)-1α (CCL3), MIP-1β (CCL4), MIP-2 (CXCL2), regulated on activation normal T cell expressed and secreted (RANTES; CCL5), TNF, and vascular endothelial growth factor (VEGF) were assayed using a Millipore Milliplex Mouse 32-Plex Cytokine- and Chemokine-Array (Millipore Sigma, #MCYTMAG-70K-PX32) by Eve Technologies (Calgary, AB, Canada). The dotted line in each graph in the results represented the limit of detection for each assay and if values were not detected, the minimum standard was utilized instead.

### Statistics

Statistics were carried out using Prism v9 (GraphPad, San Diego, CA, United States, RRID:SCR_002798). Outliers were removed using the ROUT method (Motulsky and Brown, 2006), set to a 1% threshold, in the software, as previously described (Vecchiarelli et al., 2016). All data were comparisons between two independent variables, therefore two-way analysis of variance (ANOVA)s were performed. For all ANOVA analyses, interactions and main effects were reported, and relevant comparisons were performed using Fisher’s Least Significant Difference (LSD) tests. *F*-values and *p*-values are reported in Supplementary Table 1. Data are presented as mean ± standard error of the mean (SEM). *p* < 0.05 was considered statistically significant and *p* < 0.1 was noted.

## Results

### The Fatty Acid Amide Hydrolase Single Nucleotide Polymorphism Had No Impact on Macroscopic Damage or Gut Inflammation

**FIGURE 1:**
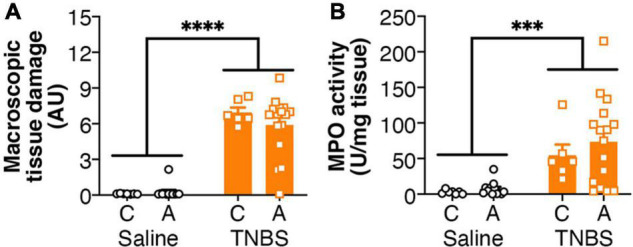
Colitis is not altered by FAAH genotype. Colitis significantly increased (A) macroscopic tissue damage and (B) MPO activity, but there was no effect of genotype, or interaction between the genotype and colitis. *n* = 6–16/group. ^∗∗∗^*p* < 0.001, ^∗∗∗∗^*p* < 0.0001, main effect of colitis. Saline = left pair, black bars with circles. TNBS = right pair, orange bars with squares. In each pair, the left bar is the C allele group (CC genotype) and the right bar is the A allele group (AC/AA genotypes).

Colitis was associated with an increased macroscopic tissue damage (Figure 1A), but this was unaffected by the genotype of the mice. Similarly, increased MPO activity was observed in mice with colitis (Figure 1B), but again there was no effect of genotype.

### Genetic Variants of Fatty Acid Amide Hydrolase Influenced Colitis-Induced Alterations in Circulating Cytokines and Chemokines

**FIGURE 2:**
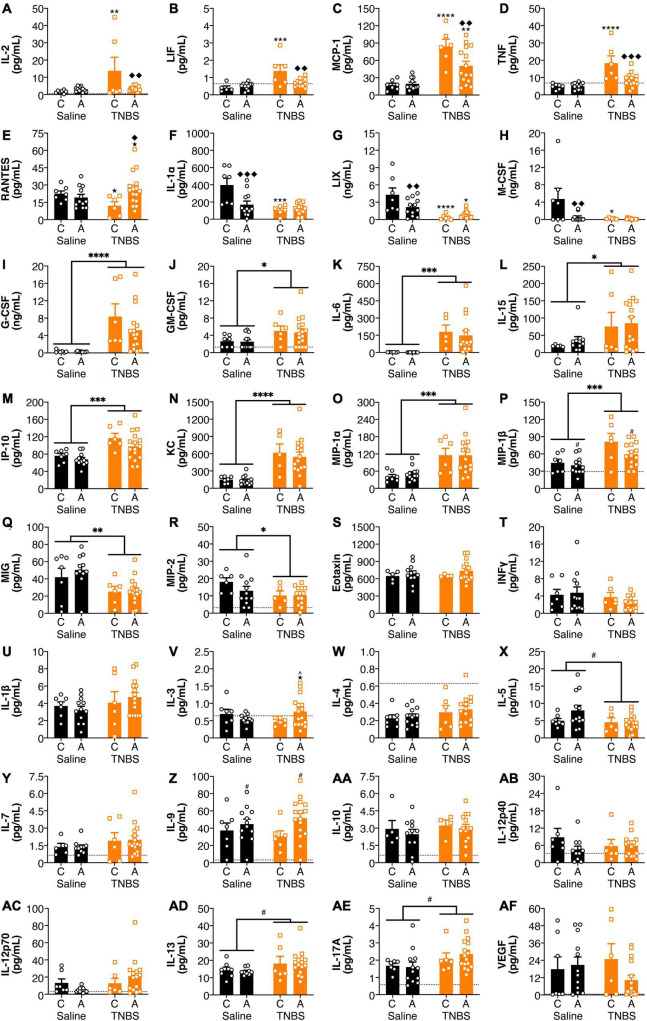
FAAH SNP altered colitis-induced changes in plasma cytokines and chemokines. Having an A allele mitigated colitis-induced increases in **(A)** IL-2, **(B)** LIF, **(C)** MCP-1, and **(D)** TNF and reductions in **(E)** RANTES. Both possessing an A allele or colitis reduced levels of **(F)** IL-1α, **(G)** LIX, and **(H)** M-CSF, but there was not an additive effect of both conditions. Colitis increased levels of **(I)** G-CSF, **(J)** GM-CSF, **(K)** IL-6, **(L)** IL-15, **(M)** IP-10, **(N)** KC, **(O)** MIP-1α and **(P)** MIP-1β and reduced levels of **(Q)** MIG and **(R)** MIP-2. There were no significant changes in **(S)** Eotaxin, **(T)** IFNγ, **(U)** IL-1β, **(V)** IL-3, **(W)** IL-4, **(X)** IL-5, **(Y)** IL-7, **(Z)** IL-9, **(AA)** IL-10, **(AB)** IL-12p40, **(AC)** IL-12p70, **(AD)** IL-13, **(AE)** IL-17A, and **(AF)** VEGF. *n* = 4–16/group. ^★^*p* < 0.1, 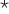
*p* < 0.05, 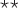
*p* < 0.01, 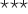
*p* < 0.001, 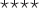
*p* < 0.0001, saline vs. TNBS of same genotype. ^*p* < 0.1, *^◆^p* < 0.05, ^◆^^◆^*p* < 0.01, ^◆^^◆^^◆^*p* < 0.001, C vs. A of same condition (saline or TNBS). ^#^*p* < 0.1, ^∗^*p* < 0.05, ^∗∗^*p* < 0.01, ^∗∗∗^*p* < 0.001, ^∗∗∗∗^*p* < 0.0001 main effect of colitis (lines) or genxotype (symbols above bars). Saline = left pair, black bars with circles. TNBS = right pair, orange bars with squares. In each pair, the left bar is the C allele group (CC genotype) and the right bar is the A allele group (AC/AA genotypes). Dotted lines indicate minimal detection threshold for the analyte and was included in graphs where values were below threshold or the threshold value was used.

Both colitis and genotype influenced plasma cytokine levels (Figure 2). For IL-2 and LIF (Figures 2A,B), inflammation-induced increases were reversed with an A genotype, whereas for MCP-1 and TNF (Figures 2C,D), colitis-induced increases were attenuated with an A genotype. For RANTES (Figure 2E), in the colitic mice, animals with an A allele had higher levels than the C allele. For IL-1α, LIX and M-CSF (Figures 2F–H), both colitis and possessing an A allele led to reductions, however, there was no interaction or additive effects between the two conditions. G-CSF, GM-CSF, IL-6, IL-15, IP-10, KC, MIP-1α, and MIP-1β (Figures 2I–P) levels were increased in animals with colitis. Conversely, MIG and MIP-2 (Figures 2Q,R) levels were decreased in animals with colitis. There were no significant changes in in Eotaxin, IFNγ, IL-1β, IL-3, IL-4, IL-5, IL-7, IL-9, IL-10, IL-12p40, 1L-12p70, IL-13, IL-17A, and VEGF levels (Figures 2S–AF) from either colitis or the FAAH SNP.

### Genetic Variants of Fatty Acid Amide Hydrolase Influenced Colitis-Induced Changes in Amygdalar Cytokines and Chemokines

**FIGURE 3:**
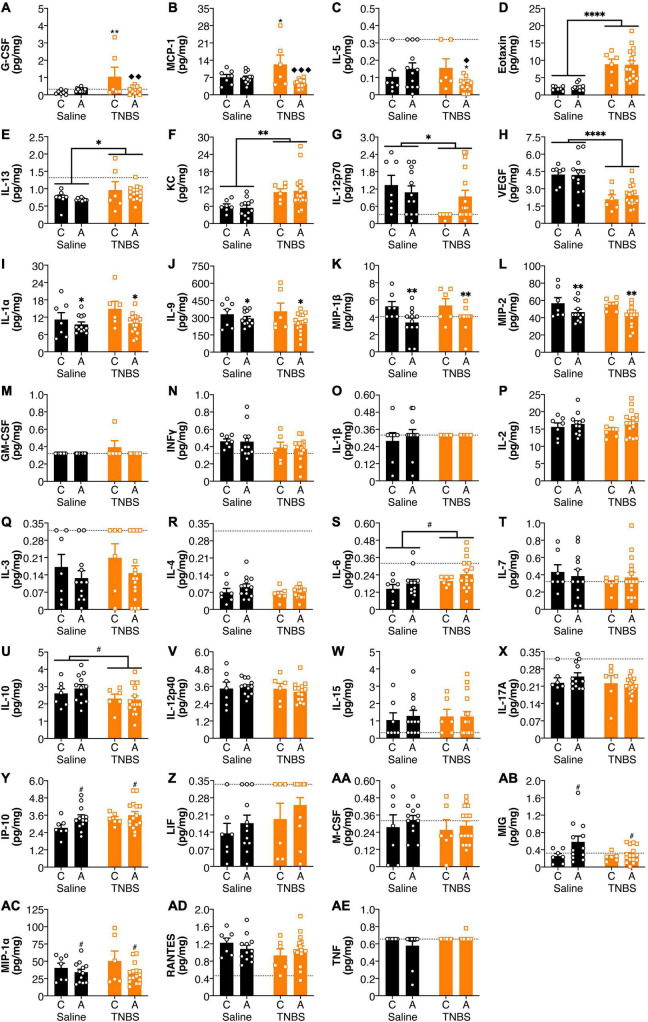
FAAH SNP altered colitis-induced changes in amygdala cytokines and chemokines. Having an A allele mitigated colitis induced increases in **(A)** G-CSF, **(B)** MCP-1, and **(C)** IL-5. Colitis increased levels **(D)** Eotaxin, **(E)** IL-13, and **(F)** KC and reduced levels of **(G)** IL-12p70 and **(H)** VEGF. Possessing an A allele led to lower levels of **(I)** IL-1α, **(J)** IL-9, **(K)** MIP-1β, and **(L)** MIP-2. There were no significant changes in **(M)** GM-CSF, **(N)** INFγ, **(O)** IL-1β, **(P)** IL-2, **(Q)** IL-3, **(R)** IL-4, **(S)** IL-6, **(T)** IL-7, **(U)** IL-10, **(V)** IL-12p40, **(W)** IL-15, **(X)** IL-17A, **(Y)** IP-10, **(Z)** LIF, **(AA)** M-CSF, **(AB)** MIG, **(AC)** MIP-1α, **(AD)** RANTES, and **(AE)** TNF. *n* = 5–16/group. 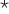
*p* < 0.05, 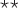
*p* < 0.01, saline vs. TNBS of same genotype. ^◆^*p* < 0.05, ^◆^^◆^*p* < 0.01, ^◆^^◆^^◆^*p* < 0.001, C vs. A of same condition (saline or TNBS). ^#^*p* < 0.1, ^∗^*p* < 0.05, ^∗∗^*p* < 0.01, ^∗∗∗∗^*p* < 0.0001 main effect of colitis (lines) or genotype (symbols above bars). Saline = left pair, black bars with circles. TNBS = right pair, orange bars with squares. In each pair, the left bar is the C allele group (CC genotype) and the right bar is the A allele group (AC/AA genotypes). Dotted lines indicate minimal detection threshold for the analyte and was included in graphs where values were below threshold or the threshold value was used.

There were effects of both colitis and the FAAH SNP on amygdala cytokine and chemokine levels (Figure 3). There was a reversal of colitis-induced changes in amygdala levels of G-CSF and MCP-1 with an A allele (Figures 3A,B). IL-5 levels (Figure 3C) were lower in the amygdala of animals possessing an A allele following colitis than either the A allele saline group or the C allele colitis group. Colitis increased amygdalar levels of Eotaxin, IL-13 and KC (Figures 3D–F); whereas levels of IL-12p70 and VEGF (Figures 3G,H) were reduced in animals with colitis. Possessing an A allele led to decreased levels of IL-1α, IL-9, MIP-1β, and MIP-2 (Figures 3I–L). There were no significant changes in the amygdala in levels of GM-CSF, IFNγ, IL-1β, IL-2, IL-3, IL-4, IL-6, IL-7, IL-10, IL-12p40, IL-15, IL-17A, IP-10, LIF, M-CSF, MIG, MIP-1α, RANTES, and TNFα (Figures 3M–AE). There were no detectable levels of LIX in the amygdala (data not shown).

## Discussion

The role of the common SNP of FAAH in inflammatory diseases is not well understood. We examined how genetic variants of FAAH could influence the peripheral and neuroinflammatory response in colitis in a recently developed mouse model expressing the human SNP of FAAH (Dincheva et al., 2015). Three days after the induction of colitis corresponds to the peak of inflammation (Elson et al., 1996), and thus we chose to examine this time point. We found that there was no effect of genotype on macroscopic tissue damage or MPO activity in the inflamed colon. Interestingly, however, genetic reduction of FAAH activity significantly attenuated or fully reversed inflammation-induced increases of plasma IL-2, LIF, MCP-1, and TNF; as well as amygdala G-CSF and MCP-1 levels. FAAH reduction in the amygdala leads to reduced IL-1α, IL-9, MIP-1β, and MIP-2 levels.

Many of the colitis-induced cytokines that we found to be influenced by the FAAH SNP (e.g., IL-2, LIF, MCP-1, and TNF), have been found to be regulated by FAAH inhibition in other studies. FAAH inhibition or boosting AEA levels inhibits IL-2 (Rockwell and Kaminski, 2004; Kaplan et al., 2005; Rockwell et al., 2008; Cencioni et al., 2010; Patsenker et al., 2015; Zajkowska et al., 2020), inhibits LIF release (Maccarrone et al., 2001; Maccarrone and Wenger, 2005), and reduces MCP-1 levels (Nakajima et al., 2006; Özdemir et al., 2014; Rivera et al., 2018; Selvaraj et al., 2019; Zhang et al., 2020; Hermes et al., 2021). There are many reports showing that FAAH inhibition or increases in AEA levels reduces TNF levels (Nakajima et al., 2006; Bátkai et al., 2007; Naidu et al., 2007; Tham et al., 2007; Roche et al., 2008; Rettori et al., 2012; Nader et al., 2014; Özdemir et al., 2014; Patsenker et al., 2015; Petrosino et al., 2018; Rivera et al., 2018; Selvaraj et al., 2019; Shang et al., 2020). Following a wide range of inflammatory stimuli, FAAH inhibition or increasing AEA levels also leads to a reduction in IFNγ, IL-1β, IL-6, IL-8, among others (Nakajima et al., 2006; Rettori et al., 2012; Özdemir et al., 2014; Patsenker et al., 2015; Chiurchiù et al., 2016; Petrosino et al., 2018; Rivera et al., 2018; Selvaraj et al., 2019; Zhang et al., 2020). This is, to the best of our knowledge, the first report of FAAH/AEA’s influence on G-CSF levels. Our work is consistent with these previous reports and is the first to report an immunomodulatory effect of this SNP, which reduces FAAH activity, including in the context of central cytokine changes in a model of peripheral inflammatory disease. However, it is unclear why reducing FAAH activity does not attenuate colitis-induced increases in IL-6, given what others have shown. We only measure one time point, and it is possible that without a time course analysis, we are missing subtle dynamics as to the effects of both colitis, FAAH genotype and their combination on cytokine expression levels, both peripherally and centrally.

Intriguingly, our results show no changes in colonic macroscopic tissue damage and MPO activity, but do show the ability of the FAAH SNP to attenuate some changes in plasma cytokines/chemokines. Therefore, it seems unlikely that the attenuation of some plasma cytokines altered with colitis in mice with an A SNP is due to changes in their production in the colon. As such, it seems likely that the elevated levels of anandamide seen in this FAAH SNP may act distal to the colon, likely directly on immune cells in the circulation or resident in the brain, to dampen the release of immunomodulatory cytokines.

Given that we have previously demonstrated that elevations of FAAH within the amygdala following the induction of colitis were associated with the development of anxiety (Vecchiarelli et al., 2021), which in peripheral inflammatory contexts is known to be driven by inflammatory cytokines within the amygdala proper (Chen et al., 2013), these data also suggest that a potential mechanism by which elevated AEA signaling may be able to dampen inflammation-associated anxiety is via a suppression of inflammatory cytokine levels within the amygdala.

FAAH reductions ameliorate colitis-induced increases in circulating IL-2, LIF, MCP-1, and TNF, which implies that FAAH activity 3 days after TNBS administration may contribute to T cell differentiation, proliferation and migration, as well as monocyte trafficking in this model, perhaps by altering levels of AEA. *Faah* expression has been shown to be increased in the proximal colon in this model of inflammation (Storr et al., 2008a), indicating that colitis may alter FAAH at this time point, thus suggesting that the loss of function FAAH SNP we are exploring in the current study may confer some immunomodulatory benefit by reducing the impacts of this upregulation of FAAH. We also see that at baseline FAAH activity indirectly regulates other inflammatory molecules in the amygdala, particularly IL-9, MIP-1β, and MIP-2, which stimulate mast cells and neutrophils (Ren et al., 2010; Rojas-Zuleta and Sanchez, 2017), as well as IL-1α, contributing to our understanding of AEA as a potential modulator of inflammatory responses. To the best of our knowledge, we are the first to demonstrate that FAAH inhibition regulates IL-1α, IL-9, MIP-1β, and MIP-2 levels.

These effects on cytokine levels are perhaps not only due to alterations in the levels of AEA caused by the FAAH SNP. FAAH hydrolyzes other fatty acid ethanolamides including palmitoylethanolamide (PEA) and oleoylethanolamide (OEA), which have anti-inflammatory effects (Pontis et al., 2016). OEA inhibits MCP-1 levels (Montecucco et al., 2015; Antón et al., 2017; Zhao et al., 2018) and also reduces TNF levels (Sayd et al., 2014; Chen et al., 2015; Xu et al., 2016; Antón et al., 2017). PEA reduces MCP-1 levels (D’Aloia et al., 2021; Fusco et al., 2021) and TNF levels (Costa et al., 2008; De Filippis et al., 2009; Hoareau et al., 2009; Cerrato et al., 2010; Di Paola et al., 2012, 2016; Sayd et al., 2014; Impellizzeri et al., 2015; Rahimi et al., 2015; Orefice et al., 2016; Britti et al., 2017; Gugliandolo et al., 2017; D’amico et al., 2019; Puglia et al., 2020; Ardizzone et al., 2021; D’Aloia et al., 2021). PEA reduces inflammation in human colonic tissue (from patients with IBD, colon cancer and appendicitis), including levels of IL-6, IL-8, IL-17A, MCP-1, and GM-CSF (Couch et al., 2017). There are no reported effects of PEA or OEA on IL-2, LIF, or G-CSF, although this does not rule out any effects in our model in these cytokines being attributed to either PEA or OEA. Given that PEA and OEA are not only metabolized by FAAH, but also have immunomodulatory effects, it is possible that the effects we observed are not limited to AEA and the endocannabinoid system, but are a concerted network effect produced by the elevations of all of these fatty acid amides acting in tandem.

In the human literature, there are very few reports examining the relationship between the FAAH SNP (C385A) and inflammatory diseases. In one study, in MS patients who received IFNγ (resulting in a condition called flu-like syndrome), possessing the A allele of this SNP did not alter any of the syndrome symptoms measured, including general malaise, muscle pain, chills, weakness and the need to take non-steroidal anti-inflammatory drugs (Buttari et al., 2017). Additionally, one report investigated this SNP in relation to IBD (Storr et al., 2009). They find that there are no differences in the frequency, and thus potentially, susceptibility, of this genotype among controls, or patients with ulcerative colitis or Crohn’s disease (for which TNBS administration is a model) (Storr et al., 2009). They do show, however, that in patients with Crohn’s disease, being homozygous for the A allele leads to a more severe phenotype, including fistulas and extra-intestinal manifestations (Storr et al., 2009). In ulcerative colitis patients, being homozygous for the A allele is associated with an earlier disease onset (Storr et al., 2009). Interestingly, patients with type two diabetes carrying an A allele (C385A) have higher TNF levels (de Luis et al., 2010), indicating for some diseases, this allele may not always confer protective effects. Inflammation can increase AEA levels (Liu et al., 2003; Doenni et al., 2016; Grill et al., 2019), and therefore, it is possible that in the context of a reduction in FAAH activity, as seen in this genotype, there is even more AEA. Greater AEA and other mediators may act on the transient receptor potential vanilloid type 1 (TRPV1) or other receptors to drive inflammation or inflammatory damage. While we did not see any exacerbation of the primary disease pathology itself by the FAAH SNP, we did look at an early time point to align with the peak of the acute phase of colitis, so it remains possible that this SNP could have deleterious effects on the disease progression under more long-term or chronic conditions.

In summary, in mice with acute colitis, increased plasma and amygdala cytokine levels are regulated by the activity of FAAH. Reductions in FAAH activity are able to reverse some of the peripheral and central inflammatory changes, particularly in those that drive monocyte and CD4^+^ T cell activation and migration. Since there were no effects of the genotype on colitis, these effects are likely mediated by the actions of elevated AEA (or other fatty acid amides) signaling to circulating or resident immune cells. Our data will inform future approaches to the personalized treatment of individuals with FAAH SNPs and may contribute to an understanding of disease outcome and progression, potentially providing treatment insights for these individuals.

## Data Availability Statement

The raw data supporting the conclusions of this article will be made available by the authors, without undue reservation.

## Ethics Statement

The animal study was reviewed and approved by the Health Sciences Animal Care Committee of the University of Calgary and followed guidelines from the Canadian Council on Animal Care.

## Author Contributions

HV, CK, QP, KS, and MH designed the experiments. HV, RA, CH, VC, MM, and AN performed the experiments. FL contributed experimental tools. HV analyzed the data and prepared the figures. HV and MH wrote the manuscript with input from all authors. QP, KS, and MH obtained funding for the work and provided oversight and supervision.

## Conflict of Interest

KS has been an advisor to Arena Pharmaceuticals and has received a research grant from Abalone Inc. MH was a scientific advisor for Sophren Therapeutics, Jazz Pharmaceuticals and Lundbeck. The remaining authors declare that the research was conducted in the absence of any commercial or financial relationships that could be construed as a potential conflict of interest.

## Publisher’s Note

All claims expressed in this article are solely those of the authors and do not necessarily represent those of their affiliated organizations, or those of the publisher, the editors and the reviewers. Any product that may be evaluated in this article, or claim that may be made by its manufacturer, is not guaranteed or endorsed by the publisher.
